# The impact of the mode of survey administration on estimates of daily smoking for mobile phone only users

**DOI:** 10.1186/s12874-017-0342-4

**Published:** 2017-04-20

**Authors:** Joseph Hanna, Damien V. Cordery, David G. Steel, Walter Davis, Timothy C. Harrold

**Affiliations:** 1 NSW Biostatistics Training Program, NSW Ministry of Health, 73 Miller Street, Locked Mail Bag 961, North Sydney, NSW 2059 Australia; 2 Centre for Epidemiology and Evidence, NSW Ministry of Health, 73 Miller Street, North Sydney, Australia; 3 National Institute for Applied Statistics Research Australia, University of Wollongong, Wollongong, Australia

**Keywords:** CATI survey, Self-administered survey, Daily smoking prevalence, Combining surveys

## Abstract

**Background:**

Over the past decade, there have been substantial changes in landline and mobile phone ownership, with a substantial increase in the proportion of mobile-only households. Estimates of daily smoking rates for the mobile phone only (MPO) population have been found to be substantially higher than the rest of the population and telephone surveys that use a dual sampling frame (landline and mobile phones) are now considered best practice. Smoking is seen as an undesirable behaviour; measuring such behaviours using an interviewer may lead to lower estimates when using telephone based surveys compared to self-administered approaches. This study aims to assess whether higher daily smoking estimates observed for the mobile phone only population can be explained by administrative features of surveys, after accounting for differences in the phone ownership population groups.

**Methods:**

Data on New South Wales (NSW) residents aged 18 years or older from the NSW Population Health Survey (PHS), a telephone survey, and the National Drug Strategy Household Survey (NDSHS), a self-administered survey, were combined, with weights adjusted to match the 2013 population. Design-adjusted prevalence estimates and odds ratios were calculated using survey analysis procedures available in SAS 9.4.

**Results:**

Both the PHS and NDSHS gave the same estimates for daily smoking (12%) and similar estimates for MPO users (20% and 18% respectively). Pooled data showed that daily smoking was 19% for MPO users, compared to 10% for dual phone owners, and 12% for landline phone only users. Prevalence estimates for MPO users across both surveys were consistently higher than other phone ownership groups. Differences in estimates for the MPO population compared to other phone ownership groups persisted even after adjustment for the mode of collection and demographic factors.

**Conclusions:**

Daily smoking rates were consistently higher for the mobile phone only population and this was not driven by the mode of survey collection. This supports the assertion that the use of a dual sampling frame addresses coverage issues that would otherwise be present in telephone surveys that only made use of a landline sampling frame.

**Electronic supplementary material:**

The online version of this article (doi:10.1186/s12874-017-0342-4) contains supplementary material, which is available to authorized users.

## Background

Over the past decade, there have been substantial changes in landline and mobile phone ownership, with most nations observing declines in landline ownership and a corresponding increase in the proportion of mobile phone only (MPO) households [1–3]. In Australia, these changes in phone ownership have not been uniform across all population groups, with many harder to reach groups, such as males, younger people, recent migrants, renters and people from a low socioeconomic background less likely to own a landline telephone [1, 4, 5]. For this reason, health surveys of the general population that only use a landline phone number sampling frame no longer have adequate population coverage to produce unbiased estimates of health behaviours [4, 6]. With the decreasing coverage of landline phone number sampling frames, it has become necessary to use dual sampling frames, which use mobile and landline phone numbers and accounts for the overlapping chance of selection, or alternatively, use non-telephone based survey approaches in order to ensure that representative estimates of health behaviours can be produced. There has been substantial work undertaken to implement dual sampling frames for health surveys and to determine whether dual sampling frames are able to correct for biases in health behaviours estimates. One key population group that appears to be quite distinct from others is the MPO population [6, 7].

Prevalence estimates for a number of health indicators, including smoking, alcohol consumption and adequate physical activity, have been found to be much higher for the MPO population compared with the landline-accessible population [6]. While some of these disparities had been explained by differences in population structure, smoking estimates were found to be persistently higher for the MPO population [6–8]. Given the emerging use of dual frame telephone surveys, higher smoking estimates obtained for the MPO population warrants further investigation [8].

In recognition of the increasing size of the MPO population, Livingston et al. have recommended that telephone surveys allow for a larger mobile subsample to ensure that the growing population of mobile-only users is properly represented in survey estimates [8], however this work predominantly focussed on measures of alcohol consumption.

There is substantial evidence that survey respondents are more likely to under-report undesirable behaviours when participating in interviewer-directed surveys (face-to-face and telephone interviewing modes) compared to self-administered surveys (such as self-complete questionnaires) [9]. Compared to self-administered surveys, interview-administered survey results were more likely to be biased towards more socially desirable responses with regards to health-related lifestyle questions [10, 11]. Therefore, further work is required to identify whether it is the mode of survey collection which influences estimates for the MPO population, particularly for smoking.

This study aims to identify whether higher smoking estimates for the MPO population can be explained by the mode of data collection for a survey, after accounting for differences in the population structure of each phone ownership population. This paper focusses on comparing daily smoking estimates from a CATI dual-frame survey with those arising from a self-administered survey for NSW, as well as a brief examination of the results for key demographic strata. A comparison of estimates from the different phone ownership groups will also be made.

## Methods

### Data sources

Data from the New South Wales Population Health Survey (NSWPHS) [12] and the National Drug Strategy Household Survey (NDSHS) were obtained for this study [13].

New South Wales (NSW) is the most populous state in Australia with an estimated population of 7.41 million in June 2013 including both highly urbanised and rural areas, and accounts for approximately one third of the Australian population [14]. At June 2013, 21% of the total Australian adult population were estimated to be mobile phone only users and the majority of these people were aged 18–34 [1]. The NSWPHS, administered by the Centre for Epidemiology and Evidence, NSW Ministry of Health, sampled respondents living in private households using CATI software to sample NSW residents according to Local Health District boundaries [15]. In 2013, an overlapping dual-frame was adopted using a sample of landline and mobile phone numbers, with a target of 30% of all interviews completed on a mobile phone, with the remainder completed on a landline telephone. A stratified two-stage cluster sample design was used for the landline frame, using simple random sampling to select clusters (household telephone numbers) within Local Health District strata and to select one household resident from the selected households using the Kish Grid [16]. A simple random sample of mobile phone numbers was selected to obtain an additional sample of adult respondents. Interviews were conducted between February and December 2013. A dual-frame weighting approach was developed to allow for the different probabilities of selection for landline only, dual-phone and mobile-only users [17], by accounting for the overlapping probability of selection for dual-phone type owners. The survey was also weighted to match Local Health District, age group and sex population estimates from the Australian Bureau of Statistics (ABS) 2013 mid-calendar year population [18].

The NDSHS, administered by the Australian Institute of Health and Welfare, was based on private dwelling households across Australia, sampling respondents aged 12 years or older [13]. Private dwellings such as hotels, motels and boarding houses were excluded from the sample as were institutional settings. A multi-stage stratified random sample was used where each state was divided into two strata; capital city and rest of state [19]. Smaller geographical areas, Statistical Area 1 (SA1) in the capital city strata and Statistical Area 2 (SA2) in the rest of state strata, were selected with probability proportional to size (based on the total number of households), with households sampled systematically within each smaller area [20]. The target respondent within each household was selected using the next birthday method. The survey was administered between 31 July and 1 December 2013, with respondents completing a de-identified paper copy of the questionnaire without an interviewer present. Population estimates used for weighting were based on the age and sex profile of each stratum using the June 2012 ABS estimated resident population. All analyses which follow are for respondents aged 18 years or older and were restricted to NSW residents.

NSWPHS data was accessed through Secure Analytics for Population Health Research and Intelligence. NDSHS data was accessed through the Australian Data Archive.

### Data preparation

Data from the NSWPHS and the NDSHS was prepared to facilitate combined analyses by appending data from the NDSHS to the NSWPHS, with analyses restricted to respondents 18 years and older. A review of demographic and geographical variables common to the two surveys was undertaken to ensure that definitions were standardised prior to any analysis. Variables in both surveys were harmonised to a common standard, except where this was not possible, such as socio-economic status and remoteness status. For the NDSHS, these concepts were mapped to the data via a concordance file at the SA1-level. For the NSWPHS, these concepts were mapped to the data via a concordance file at the postcode level. SA1 and postcode represented the finest spatial boundaries available for both surveys, with postcodes generally representing larger spatial regions on average compared to SA1s in Australia. Socio-economic status (SES) was defined according to the Index of Relative Socio-Economic Advantage and Disadvantage [21]. Remoteness was defined according to the Accessibility and Remoteness Index of Australia [22]. Phone ownership status was derived from information about the number of landline telephones and mobile phones the respondent personally owned on the NSWPHS and by two questions on telephone ownership on the NDSHS. The NDSHS included a small proportion of respondents who had no telephone and these were excluded from the analysis. Other variables which could not be harmonised were not used in the analysis [23]. Therefore, we were not able to include income as a covariate, and used socioeconomic and remoteness status of the area as a proximate measure. Although there were slight differences in the way that the smoking status question was asked between the surveys, we were able to identify a common response category, which was daily use (see Additional file 1). Stratification variables for the two surveys were treated as distinct strata [24]. Further, as the primary sampling unit for each survey was different (SA1 and SA2 in the NDSHS; households in the NSWPHS); a new cluster variable was derived to account for this. Weights for both surveys were adjusted to represent 2013 population counts using an overall adjustment factor of *N*_2_/(*N*_1_ + *N*_2_) where N_2_ is the 2013 population count and N_1_ is the 2012 population count [23]. This adjustment ensures that the sum of the weights for the combined sample is equal to the 2013 population count and effectively adjusts for the two samples covering the same population and that they contribute approximately equally. All statistical analysis was performed on the combined data from the NSWPHS and the NDSHS.

### Statistical methods

The independent variable of interest in our analyses was phone ownership status, which was made up of three categories, defined as: mobile-only, dual phone, and landline-only. The dependent variable is daily smoking status. The other covariates considered were collection mode (self-administered; CATI), sex (female; male), age group (18–24; 25–34; 35–44; 45–54; 55–64; 65+), remoteness (metropolitan; rural/regional), socio-economic status (5 quintiles), country of birth (Australia; other) and education (trade certificate or higher; no qualifications).

Daily smoking prevalence estimates, odds ratios, and confidence intervals were estimated using SAS 9.4. Odds ratios were computed using SAS’s SURVEY LOGISTIC procedure and prevalence estimates were obtained using the SURVEY MEANS procedure. The Taylor series linearisation method was used to estimate the variance of prevalence estimates and model parameter estimates [24].

### Sensitivity analyses

As data from the NDSHS was only collected during the second half of 2013, a sensitivity analysis was performed by restricting data from the NSWPHS to the same period and comparing our findings from the full dataset to the restricted data. Similar results would confirm the validity of the analysis and warrant no further investigation of a possible seasonal effect in the data.

Further, to compare the results with a more precise weighting adjustment, the weighting adjustment factor of *N*_2_/(*N*_1_ + *N*_2_) was also applied for each age and sex stratum as a second sensitivity analysis [24]. While an overall weighting adjustment is usually recommended [23], further investigation of the impact of any adjustment factor was warranted to ensure that our findings were robust to one of the key decisions made when combining data from the two surveys.

## Results

### Respondent profile

A total of 12,751 respondents from the NSWPHS were aged 18 years or older and a total of 6,009 from the NDSHS were NSW residents aged 18 years or older who owned a telephone. Response rates were 30% for the NSWPHS and 32.7% for the NDSHS using the American Association for Public Opinion Research defined Response Rate 3 [25]. Mobile-only respondents constituted 9.6% of the NSWPHS sample under consideration, 77.9% were dual phone respondents, and 12.5% were landline-only respondents. By contrast, mobile-only respondents constituted 20.5% of the NDSHS sample under consideration, 54.3% were dual phone respondents and 25.2% were landline-only respondents. The distribution of respondent characteristics across the two surveys is shown in Table 1. The sex distribution for the mobile-only respondents was similar to the other phone ownership groups. Mobile-only respondents were more likely to be younger and had the largest proportion of respondents in the 25–34 years category.

**Table 1:** Selected characteristics of the two survey populations by frame (unweighted)

		CATI %				Self-administered %				Combined Sample %			
Variable	Category	Mobile only	Dual phone	Landline only	Total	Mobile only	Dual phone	Landline only	Total	Mobile only	Dual phone	Landline only	Total
Sex	Male	50.3	40.0	43.1	41.5	43.2	46.7	42.4	45.1	46.9	41.6	42.7	42.6
	Female	49.7	60.0	56.9	58.5	56.8	53.3	57.6	54.9	53.1	58.4	57.3	57.4
Age	18-24	17.5	6.3	0.4	6.7	12.1	3.9	7.2	6.7	14.9	5.7	3.6	6.7
	25-34	33.4	9.5	1.3	10.8	28.6	8.2	10.1	13.2	31.1	9.2	5.4	11.6
	35-44	20.1	12.3	2.7	11.9	23.5	15.8	16.4	17.4	21.8	13.1	9.1	13.7
	45-54	13.6	18.4	9.4	16.8	15.9	18.1	16.1	16.8	14.7	18.3	12.6	16.8
	55-64	10.1	23.4	17.4	21.3	11.4	20.9	17.5	17.6	10.7	22.8	17.4	20.1
	65+	5.3	30.1	68.7	32.5	8.4	33.1	32.8	28.3	6.8	30.8	51.9	31.2
Remoteness	Metropolitan	80.0	59.9	44.6	60.1	72.1	73.5	73.3	73.4	76.1	63.1	58.0	64.4
	Rural/regional	20.0	40.1	55.4	39.9	27.9	26.5	26.7	26.6	23.9	36.9	42.0	35.6
SES	1st Quintile (lowest SES)	16.5	18.6	27.3	19.4	19.8	15.4	20.3	17.7	18.1	17.8	24.0	18.9
	2nd Quintile	18.0	26.1	31.3	25.9	21.8	17.8	18.9	19.0	19.9	24.2	25.5	23.7
	3rd Quintile	21.2	21.8	20.4	21.6	15.3	15.8	16.0	15.8	18.3	20.4	18.4	19.7
	4th Quintile	15.1	10.8	7.7	10.8	19.8	21.4	20.3	20.7	17.4	13.2	13.6	13.9
	5th Quintile (highest SES)	29.1	22.7	13.3	22.3	23.3	29.7	24.6	26.8	26.3	24.4	18.6	23.7
COB	Australia	56.4	71.5	77.6	70.6	72.3	73.5	68.1	68.9	64.1	72.0	73.2	70.1
	Other	43.6	28.5	22.4	29.4	27.7	26.5	31.9	31.1	35.9	28.0	26.8	29.9
Education	Trade certificate or higher	65.3	61.9	38.2	59.3	73.6	72.1	59.8	69.1	69.3	64.3	48.3	62.4
	No qualifications	34.7	38.1	61.8	40.7	26.4	27.9	40.2	30.9	30.7	35.7	51.7	37.6

### Prevalence estimates

Daily smoking estimates by key demographic characteristics, collection mode and phone ownership status are presented in Table 2. The overall daily smoking estimate from the NSWPHS was similar to the NDSHS estimate (around 12% of the population reported daily smoking). Daily smoking estimates were similar for males and females for the two surveys. Although estimates by age group differed slightly between the surveys, a similar risk profile was apparent for each collection method, preserving disparities separately identifiable in each survey, such as higher smoking rates for males, people living in rural areas, and people living in the first socio-economic quintile. While daily smoking estimates for the MPO population were higher in the NSWPHS (20.3%, 95% CI: 17.5-23.1) than the NDSHS (17.8%, 95% CI: 15.1-20.5), estimates from both surveys were higher than all other phone ownership groups across the majority of subgroups.

**Table 2:** Daily smoking estimates by selected characteristics and collection frame (weighted)

		CATI % (95% CI)				Self-administered % (95% CI)				Combined Sample % (95% CI)			
Variable	Category	Mobile only	Dual phone	Landline only	Total	Mobile only	Dual phone	Landline only	Total	Mobile only	Dual phone	Landline only	Total
Sex	Male	24.9 (20.6, 29.1)	11.6 (10.2, 13.1)	12.7 (9.2, 16.1)	15.1 (13.6, 16.6)	21.7 (17.3, 26.1)	10.5 (8.5, 12.5)	17.1 (13.2, 21.0)	14.0 (12.2, 15.7)	23.6 (20.5, 26.6)	11.1 (9.9, 12.3)	15.6 (12.8, 18.4)	14.6 (13.4, 15.7)
	Female	15.1 (11.6, 18.6)	8.4 (7.3, 9.5)	6.6 (4.1, 9.0)	9.4 (8.4, 10.5)	14.1 (11.2, 17.0)	9.9 (8.1, 11.7)	8.4 (6.2, 10.5)	10.6 (9.2, 12.0)	14.6 (12.3, 17.0)	9.0 (8.0, 9.9)	7.8 (6.2, 9.5)	10.0 (9.1, 10.9)
Age	18-24	18.9 (12.3, 25.5)	9.4 (6.5, 12.3)	12.7 (0.0, 37.8)	12.7 (9.6, 15.7)	16.0 (9.3, 22.7)	9.0 (3.3, 14.7)	7.5 (0.5, 14.5)	11.3 (7.6, 15.0)	17.7 (12.9, 22.4)	9.3 (6.6, 11.9)	7.7 (1.0, 14.5)	12.0 (9.7, 14.4)
	25-34	18.3 (13.5, 23.1)	12.3 (9.4, 15.2)	23.5 (0.0, 53.3)	15.2 (12.5, 17.9)	16.4 (11.6, 21.1)	15.0 (9.7, 20.3)	12.9 (6.5, 19.4)	14.9 (12.1, 17.7)	17.5 (14.0, 20.9)	13.2 (10.6, 15.8)	13.9 (7.4, 20.4)	15.0 (13.1, 17.0)
	35-44	17.8 (11.7, 23.9)	10.2 (8.2, 12.2)	20.1 (6.1, 34.2)	12.6 (10.3, 14.9)	17.0 (11.9, 22.1)	9.1 (6.3, 11.9)	13.7 (8.5, 18.9)	12.1 (9.6, 14.6)	17.4 (13.4, 21.5)	9.8 (8.1, 11.4)	14.7 (9.8, 19.6)	12.4 (10.7, 14.0)
	45-54	31.8 (23.3, 40.4)	13.6 (11.3, 15.9)	10.5 (4.1, 16.8)	16.2 (13.9, 18.5)	28.0 (20.3, 35.7)	12.9 (9.8, 16.0)	14.0 (9.1, 18.8)	15.3 (12.8, 17.9)	30.1 (24.3, 35.9)	13.3 (11.4, 15.2)	13.2 (9.2, 17.1)	15.8 (14.0, 17.5)
	55-64	24.7 (16.1, 33.2)	9.5 (7.7, 11.2)	17.1 (11.1, 23.2)	12.2 (10.3, 14.1)	19.0 (11.1, 26.9)	11.4 (8.5, 14.4)	16.9 (11.7, 22.0)	13.5 (11.1, 16.0)	22.3 (16.3, 28.3)	10.3 (8.7, 11.9)	17.0 (13.0, 20.9)	12.9 (11.3, 14.4)
	65+	12.5 (3.4, 21.5)	4.1 (3.2, 5.0)	5.7 (3.9, 7.5)	5.1 (4.2, 6.1)	9.1 (3.5, 14.7)	6.1 (4.4, 7.9)	10.0 (6.8, 13.1)	7.2 (5.9, 8.6)	10.8 (5.4, 16.1)	5.1 (4.1, 6.1)	7.6 (5.9, 9.4)	6.2 (5.3, 7.0)
Remoteness	Metropolitan	17.4 (14.4, 20.5)	9.4 (8.4, 10.4)	8.6 (5.9, 11.3)	11.2 (10.2, 12.3)	15.0 (11.8, 18.2)	9.3 (7.8, 10.8)	10.2 (8.0, 12.4)	10.6 (9.3, 11.8)	16.4 (14.2, 18.6)	9.4 (8.5, 10.2)	9.7 (8.0, 11.5)	10.9 (10.1, 11.7)
	Rural/regional	32.0 (25.0, 38.9)	11.4 (9.6, 13.3)	11.6 (8.2, 15.0)	15.3 (13.4, 17.3)	25.4 (21.1, 29.8)	12.7 (9.5, 15.8)	19.4 (13.5, 25.4)	17.2 (14.4, 20.0)	28.6 (24.6, 32.7)	11.9 (10.2, 13.7)	16.2 (12.4, 20.0)	16.3 (14.5, 18.0)
SES	1st Quintile (lowest SES)	29.9 (21.8, 38.0)	15.1 (12.2, 17.9)	12.5 (7.7, 17.4)	18.2 (15.4, 21.0)	37.1 (30.3, 43.9)	17.1 (13.0, 21.2)	20.8 (15.4, 26.2)	22.7 (19.1, 26.4)	33.4 (28.1, 38.7)	15.9 (13.5, 18.3)	18.1 (14.1, 22.1)	20.5 (18.2, 22.9)
	2nd Quintile	26.3 (19.6, 33.0)	13.2 (11.0, 15.4)	13.5 (8.3, 18.7)	15.7 (13.6, 17.8)	15.5 (10.3, 20.7)	13.3 (10.0, 16.6)	12.9 (8.3, 17.5)	13.0 (10.6, 15.4)	20.9 (16.6, 25.3)	13.2 (11.4, 15.1)	13.2 (9.7, 16.6)	14.4 (12.8, 16.0)
	3rd Quintile	20.2 (14.5, 25.8)	10.8 (8.9, 12.7)	7.4 (3.7, 11.1)	12.7 (10.8, 14.6)	20.5 (13.3, 27.7)	11.6 (7.7, 15.5)	15.8 (10.4, 21.3)	14.4 (11.6, 17.1)	20.3 (15.8, 24.8)	11.1 (9.2, 12.9)	12.4 (8.8, 16.0)	13.3 (11.8, 14.9)
	4th Quintile	16.0 (9.7, 22.2)	5.8 (4.0, 7.6)	3.1 (1.0, 5.3)	8.4 (6.3, 10.5)	9.4 (4.6, 14.2)	9.5 (6.8, 12.1)	10.7 (4.1, 17.3)	9.5 (7.3, 11.8)	12.6 (8.7, 16.5)	7.9 (6.1, 9.6)	9.3 (3.9, 14.6)	9.1 (7.5, 10.7)
	5th Quintile (highest SES)	13.3 (8.2, 18.4)	6.3 (5.0, 7.7)	7.7 (3.5, 11.8)	8.0 (6.5, 9.5)	8.5 (4.8, 12.2)	4.3 (2.9, 5.6)	5.2 (2.9, 7.5)	5.4 (4.2, 6.6)	11.5 (8.0, 15.0)	5.5 (4.5, 6.5)	5.9 (3.9, 8.0)	6.9 (5.8, 7.9)
COB	Australia	24.4 (20.6, 28.3)	10.4 (9.3, 11.5)	9.6 (7.2, 12.1)	13.3 (12.1, 14.4)	20.2 (16.9, 23.4)	10.6 (9.0, 12.2)	13.1 (10.4, 15.9)	13.2 (11.7, 14.6)	22.3 (19.8, 24.9)	10.5 (9.5, 11.4)	11.9 (10.0, 13.9)	13.2 (12.3, 14.1)
	Other	15.1 (10.9, 19.3)	9.0 (7.5, 10.4)	9.6 (5.5, 13.8)	10.5 (8.9, 12.0)	12.4 (8.5, 16.4)	9.0 (6.7, 11.4)	11.1 (7.8, 14.4)	10.4 (8.7, 12.1)	14.2 (11.1, 17.2)	9.0 (7.7, 10.2)	10.7 (8.0, 13.3)	10.4 (9.3, 11.5)
Education	Trade certificate or higher	16.8 (13.4, 20.2)	8.0 (7.0, 9.0)	10.6 (6.9, 14.2)	10.2 (9.1, 11.3)	14.4 (11.9, 16.8)	9.4 (7.8, 10.9)	11.8 (9.2, 14.4)	11.1 (9.8, 12.4)	15.7 (13.5, 17.8)	8.6 (7.7, 9.4)	11.5 (9.4, 13.6)	10.7 (9.8, 11.5)
	No qualifications	27.0 (22.0, 32.1)	13.6 (11.8, 15.4)	9.1 (6.5, 11.8)	15.8 (14.2, 17.5)	27.4 (21.5, 33.3)	12.3 (9.9, 14.6)	14.2 (10.4, 18.1)	15.1 (12.9, 17.3)	27.1 (23.3, 31.0)	13.1 (11.7, 14.5)	12.2 (9.7, 14.7)	15.5 (14.2, 16.9)
Total		20.3 (17.5, 23.1)	9.9 (9.0, 10.8)	9.6 (7.5, 11.7)	12.2 (11.3, 13.1)	17.8 (15.1, 20.5)	10.2 (8.8, 11.6)	12.5 (10.3, 14.7)	12.3 (11.0, 13.5)	19.2 (17.3, 21.2)	10.0 (9.2, 10.8)	11.5 (9.9, 13.2)	12.2 (11.5, 13.0)

Disparities in metropolitan and rural/regional estimates were more pronounced in the NDSHS than the NSWPHS. Daily smoking estimates by country of birth and education were similar. Daily smoking estimates for the MPO population were considerably higher than estimates from other phone ownership groups.

From the combined estimates of the two surveys, we observe that males had a higher prevalence of daily smoking (14.6%, 95% CI: 13.4-15.7) compared to females (10.0%, 95% CI: 9.1-10.9). Further, daily smoking estimates were highest in the 25–34 (15.0%, 95% CI: 13.1-17.0) and 45–54 (15.8%, 95% CI: 14.0-17.5) age categories.

### Statistical models

**Fig. 1:**
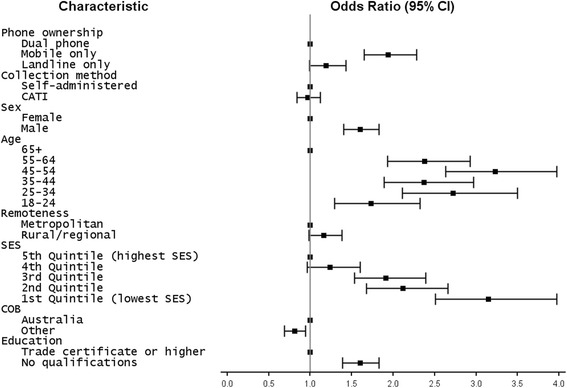
Daily smoking odds ratios and 95% confidence intervals for the final model including all covariates from the combined sample

Odds ratios for the outcome measure of daily smoking are given in Table 3. Results from the crude model (the Phone ownership effects only model) indicate that the MPO population were more likely (OR: 2.14, 95% CI: 1.84-2.49) to be daily smokers compared with the dual-phone ownership population. Interestingly, estimates for the MPO population remained identical after controlling for the collection method. Disparities in MPO population estimates of daily smoking still persisted after accounting for age group, sex, remoteness, socio-economic status, country of birth and education, while the landline-only phone ownership group remained quite similar to the dual-phone ownership group (Fig. 1).

**Table 3:** Logistic regression odds ratio estimates and 95% CIs for daily smoking from the combined sample (weighted)

Variable	Category	Phone ownership effects only	Phone ownership and survey collection method effects	Model with all covariates
Phone ownership	Dual phone	1.00	1.00	1.00
	Mobile only	2.14 (1.84, 2.49)	2.14 (1.84, 2.49)	1.94 (1.65, 2.28)
	Landline only	1.17 (0.99, 1.40)	1.17 (0.98, 1.39)	1.19 (0.99, 1.43)
Collection method	Self-administered		1.00	1.00
	CATI		0.99 (0.86, 1.14)	0.97 (0.84, 1.12)
Sex	Female			1.00
	Male			1.60 (1.40, 1.83)
Age	65+			1.00
	55-64			2.38 (1.93, 2.93)
	45-54			3.23 (2.63, 3.97)
	35-44			2.37 (1.89, 2.97)
	25-34			2.72 (2.11, 3.50)
	18-24			1.73 (1.29, 2.32)
Remoteness	Metropolitan			1.00
	Rural/regional			1.16 (0.98, 1.38)
Socio-economic status	5th Quintile (highest SES)			1.00
	4th Quintile			1.24 (0.96, 1.60)
	3rd Quintile			1.91 (1.53, 2.39)
	2nd Quintile			2.12 (1.68, 2.66)
	1st Quintile (lowest SES)			3.15 (2.51, 3.97)
Country of birth	Australia			1.00
	Other			0.81 (0.69, 0.94)
Education	Trade certificate or higher			1.00
	No qualifications			1.60 (1.39, 1.83)

### Sensitivity analyses

Restricting data from the NSWPHS data to the second half of 2013 (to match the collection period for the NDSHS) produced estimates and disparities within population sub-groups which were broadly consistent with results based on a full year of data (data not shown).

Applying the weighting adjustment to both surveys by age and sex stratum produced similar results to those obtained by using the simple weighting adjustment of *N*_2_/(*N*_1_ + *N*_2_) (data not shown).

## Discussion

This study demonstrates that CATI surveys can produce estimates that are consistent with self-administered surveys for daily smoking, not only for the total population, but also for most population sub-groups, including the MPO population. These findings were consistent even after accounting for factors such age group, sex, remoteness, socio-economic status, country of birth and education.

Higher daily smoking estimates for the MPO population were not unique to the CATI survey, with a similar pattern of estimates by phone ownership group observable for the self-administered survey. Further, this study reinforces the argument that dual sampling frames adequately address biases that arise in estimates of smoking behaviour, when compared to a self-administered survey.

Although it was anticipated that the CATI survey results were more likely to be biased towards more socially desirable responses [9–11], we noted that this effect was not observed in our study, with overall estimates for each survey almost identical, and only a 2% difference in the prevalence estimate for the MPO population between the surveys. This can be seen in Table 2 where the estimates are around 20% and 18% for the CATI and self-administered modes respectively.

The MPO population had higher odds of daily smoking even after adjusting for important demographic characteristics, which is consistent with previous research in the area [8], and has built on this research by noting that these differences have not been attributed to different collection methods. Our study has demonstrated that using a dual sampling frame (mobile and landline) for telephone interviewing can help to reduce biases arising from the declining coverage of landline phone number sampling frames, which is consistent with other findings [6, 8].

Although self-administration provides some advantages in terms of coverage for surveys of the general population, one of the primary limitations of these methods is the cost, especially compared to other interviewing modes [26]. While mobile phone interviewing is more expensive compared to landline interviewing in Australia, these differences are ameliorated when conducting national phone surveys, or studies where the target population makes up a substantial proportion of the overall population.

This study benefited from the availability of large sample sizes from the two surveys, meaning that we were able to identify whether our findings were robust to the use of different weighting adjustments, and from restricting analyses to the second half of 2013. The weighting adjustment applied ensures that the sum of the weights for the combined sample is equal to the 2013 population count and effectively adjusts for the two samples covering the same population. Our planned sensitivity analyses enabled us to determine that our model estimates were relatively robust to any potential seasonal effects arising from the differences in collection period for the surveys.

Variances for our prevalence and odds ratio estimates were estimated under the assumption that the two samples were independent. While there is the possibility of some overlap in respondents between the samples, it is negligible and will not affect the variance estimates to any meaningful degree [23]. The analysis would have benefited from harmonised SES and remoteness variables based on SA1 for all respondents. Although the SES and remoteness variables were derived at different geographic levels in the two surveys, disparities in estimates for both surveys broadly matched in terms of the direction of the association.

Although biochemical tests of tobacco use may be more precise, our study has shown estimates arising from a CATI survey were similar to those arising from a self-administered survey. Other studies have found that self-administered surveys are broadly consistent with the findings of biochemical tests [27–29]. It is also noted that responses to sensitive questions may vary depending on the specific topic [30]. Therefore, CATI responses to sensitive questions such as illicit drug use or mental health may not behave as consistently to the self-administered approach as has been observed for daily smoking estimates in this study. Further analysis and assessment of these indicators is needed in order to ascertain whether dual-frame sampling can reconcile differences in estimates of illicit drug use between CATI and self-administered surveys.

## Conclusions

We have demonstrated in this study that daily smoking estimates vary consistently across both a CATI survey and a self-administered survey. Further, we have demonstrated that higher daily smoking estimates for the MPO population are not an artefact of the dual-frame design, but have also been observed in a survey where phone ownership is not relevant to the administration of the survey. Our results provide evidence that daily smoking rates for the MPO population, while high, are not being driven by the mode of collection and lend credence to the use of dual sampling frame telephone surveys as a cost effective tool for the collection of health risk factor and behaviour information for large populations.

## Additional file

Additional file 1: Provides the questions on smoking frequency used in the NDSHS and the NSWPHS in 2013. The common response category for smoking was daily use. (DOCX 14 kb)

## Abbreviations

ABS: Australian Bureau of Statistics
CATI: Computer Assisted Telephone Interviewing
COB: Country of birth
MPO: Mobile phone only
NDSHS: National Drug Strategy Household Survey
NSW: New South Wales
NSWPHS: NSW Population Health Survey
SA1: Statistical Area Level 1
SA2: Statistical Area Level 2
SES: Socio-economic status
