# Supplementary material for: The impact of the mode of survey administration on estimates of daily smoking for mobile phone only users
Source: BMC Med Res Methodol. 2017 Apr 20;17:65. doi: 10.1186/s12874-017-0342-4 (PMC5397813; doi:10.1186/s12874-017-0342-4)
Supplement: Additional file 1: — Provides the questions on smoking frequency used in the NDSHS and the NSWPHS in 2013. The common response category for smoking was daily use. (DOCX 14 kb) [file 12874_2017_342_MOESM1_ESM.docx]

**Additional file 1**

**Survey questions: smoking frequency**

**National Drug Strategy Household Survey 2013**

**D10. How often do you now smoke cigarettes, pipes or other tobacco products?**

(Mark one response only)

- Daily
- At least weekly (but not daily)
- Less often than weekly
- Not at all, but I have smoked in the last 12 months
- Not at all and I have not smoked in the last 12 months

**NSW Population Health Survey 2013**

**SMK1. Which of the following best describes your smoking status?**

- I smoke daily
- I smoke occasionally
- I don't smoke now, but I used to
- I've tried it a few times but never smoked regularly
- I've never smoked
- Don't know
- Refused
